# Effects of lifestyle intervention on dietary intake, physical activity level, and gestational weight gain in pregnant women with different pre-pregnancy Body Mass Index in a randomized control trial

**DOI:** 10.1186/1471-2393-14-331

**Published:** 2014-09-24

**Authors:** Amy Leung Hui, Lisa Back, Sora Ludwig, Phillip Gardiner, Gustaaf Sevenhuysen, Heather J Dean, Elisabeth Sellers, Jonathan McGavock, Margaret Morris, Depeng Jiang, Garry X Shen

**Affiliations:** Departments of Internal Medicine, University of Manitoba, Room 801D, 715 McDermot Ave, Winnipeg, MB R3E 3P4 Canada; Kinesiology, University of Manitoba, Winnipeg, MB Canada; Human Nutritional Sciences, University of Manitoba, Winnipeg, MB Canada; Department of Pediatrics and Child Health, University of Manitoba, Winnipeg, MB Canada; Obstetrics and Gynecology, University of Manitoba, Winnipeg, MB Canada

**Keywords:** Pregnancy, Gestational diabetes, Nutrition, Pregnancy weight gain

## Abstract

**Background:**

The objectives of this study were to assess the efficacy of lifestyle intervention on gestational weight gain in pregnant women with normal and above normal body mass index (BMI) in a randomized controlled trial.

**Methods:**

A total of 116 pregnant women (<20 weeks of pregnancy) without diabetes were enrolled and 113 pregnant women completed the program. Participants were randomized into intervention and control groups. Women in the intervention group received weekly trainer-led group exercise sessions, instructed home exercise for 3-5-times/week during 20-36 weeks of gestation, and dietary counseling twice during pregnancy. Participants in the control group did not receive the intervention. All participants completed a physical activity questionnaire and a 3-day food record at enrolment and 2 months after enrolment.

**Results:**

The participants in the intervention group with normal pre-pregnancy BMI (≤24.9 kg/M2, n = 30) had lower gestational weight gain (GWG), offspring birth weight and excessive gestational weight gain (EGWG) on pregnancy weight gain compared to the control group (n = 27, p < 0.05). Those weight related-changes were not detected between the intervention (n = 27) and control group (n = 29) in the above normal pre-pregnancy BMI participants. Intervention reduced total calorie, total fat, saturated fat and cholesterol intake were detected in women with normal or above normal pre-pregnancy BMI compared to the control group (p < 0.05 or 0.01). Increased physical activity and reduced carbohydrate intake were detected in women with normal (p < 0.05), but not above normal, pre-pregnancy BMI at 2 months after the onset of the intervention compared to the control group.

**Conclusion:**

The results of the present study demonstrated that the lifestyle intervention program decreased EGWG, GWG, offspring birth weight in pregnant women with normal, but not above normal, pre-pregnancy BMI, which was associated with increased physical activity and decreased carbohydrate intake.

**Trial registration:**

NCT00486629

## Background

Obesity has been recognized as a health issue, which increases risk for several common chronic diseases [1–3]. The guidelines of the Canadian Medical Association Institute for the management and prevention of obesity have recommended the measurement of both body mass index (BMI) and waist circumference to assess the level and distribution of adiposity in adults [4]. BMI was calculated as weight in kilograms divided by height in meters squared. Health Canada defines underweight as a BMI less than 18.5 kg/m [2], normal weight as a BMI of 18.5-24.9, overweight as a BMI of 25.0-29.9, class I obesity as a BMI of 30.0 - 34.9, class II obesity as a BMI of 35.0 -39.9, and class III obesity as a BMI ≥ 40.0 [1]. In Canada, it was estimated that approximately 8.6 million of adults with age >18 years were overweight and 5.5 million were obese in 2005 [5]. The rising prevalence of obesity has increased the percentage of obesity in women at childbearing age in Canada. The 2006-2007 Canadian Maternal Experience Survey estimated that approximately 23% and 18% of the women began their pregnancy as overweight or obese [6]. Pre-pregnancy BMI ≥25 or above normal BMI increases the risk of poor outcomes of pregnancy including gestational diabetes, preeclampsia, hypertension and cesarean section [7].

In 2009, the Institute of Medicine (IOM) revised the 1990 guidelines of recommended weight gain during pregnancy in response to the worldwide epidemic of obesity and the demand to reduce obesity [7]. The guidelines have been endorsed by the American College of Obstetricians and Gynecologists [8] and the Health Canada [9]. Behavioral interventions such as weight awareness and dietary pattern improvement may mitigate the risks of pregnancy complications. Several studies examined the impact of lifestyle interventions (dietary intervention with or without added physical activity) on excessive gestational weight gain (EGWG) using the IOM 2009 guidelines. The results of those studies, either randomized controlled trials (RCT) or clinical studies, were not homogenous [10–19]. We hypothesize that normal weight and above normal weight pregnant women may have different responses to a lifestyle intervention in terms of gestational weight gain (GWG).

In order to test this hypothesis, we examined the impact of a lifestyle intervention program on pregnant women in normal and above normal pre-pregnancy BMI categories. EGWG, physical activity levels, dietary intake were compared between the control and intervention groups in each BMI category via a RCT.

## Methods

### Subjects

**Figure 1:**
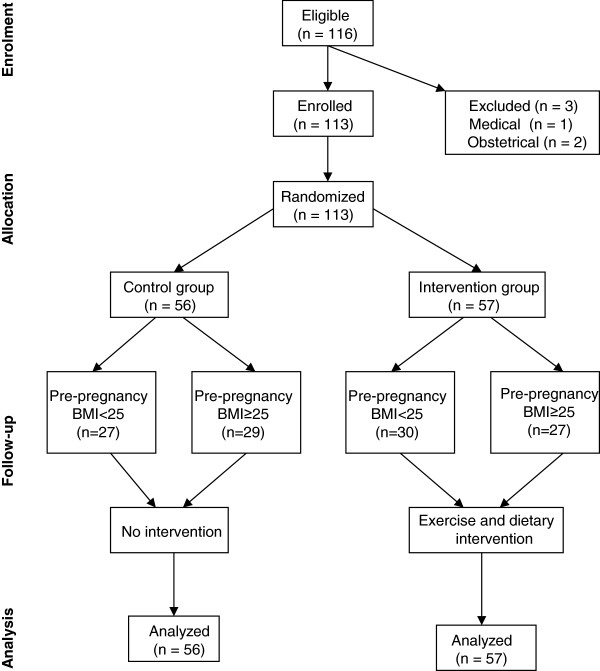
**Consort flow chart of the study.**

This study recruited 116 pregnant women who lived in Winnipeg, Manitoba between May 2009 and December 2011. This sample size was based on two previous studies that using Pre-pregnancy BMI subgroups and detected significant gestational weight gain difference between two BMI groups [13, 16]. Inclusion criteria were: less than 20 weeks of pregnancy, no existing diabetes during pregnancy and signed consent form. These participants were recruited from prenatal classes or community clinics through posters or local newspaper advertisements in Winnipeg. The study protocol and consent form were approved by the Research Ethics Board of the University of Manitoba. Three applicants were excluded from the study because of the existence of medical or obstetric contraindication for exercise during pregnancy. One hundred and thirteen eligible participants were randomized into control or intervention group (Figure 1). Randomization was performed using a computer-generated randomization allocation table by a staff member without involvement in the study design. After randomization, participants received a sealed envelope labelled with the assigned randomization number, which contained instructions for participants. The nature of the study meant that participants and study staff were not blinded to the types of interventions. None of the participants discontinued during the participation. No complain to the program was reported by the participants.

### Intervention program

#### Instructed exercise

Participants in the intervention group received community-based weekly exercise program which was developed in our previous studies [19, 20]. The exercise program included mild-to-moderate aerobic exercise, stretching, and strength exercise, and was delivered in weekly group exercise class or a DVD format to assist home exercise. Participants were encouraged to exercise for 3-5 times a week, 30-45 minutes/time, including attending group exercise class or following the exercise DVD instruction at home. The exercise intervention period was from 20-26 gestational weeks to 36 gestational weeks. Participants kept a logbook on their exercise activities as a motivator for exercise. Attendance less than 3 times at the group exercise, showed no interest to exercise at home or no record of exercise in logbook were considered as withdraw from the study.

#### Dietary intervention

Participants in the intervention group received one-on-one private dietary consultation at baseline and at two months after. The dietary consultation was performed using a Food Choice Map (FCM) software. The FCM has been proved to be a valid tool for assessing dietary intake [21]. During the consultation, participants recalled their food intakes in a typical week. Participants and dietitians jointly placed food stickers on a magnetic board. The nutritional information on the food sticker, including food items, portion sizes, the frequency of each food, was scanned into the computer at the end of each interview [22]. Daily calorie intake and macronutrients were analyzed instantly. Nutritional recommendations were based on the dietary intake analysis and Health Canada guidelines for food intake in pregnancy [23, 24] with considerations on personal food preference, food beliefs, and food budgeting. Weight gain goal was discussed and emphasized through consultation. FCM is an effective way to identify factors that are relevant to a particular health behavior in a population under investigation. Such a tool allows the participants to comment on all the foods that she consumed in real life, without missing or neglecting certain foods cognitively. Results from this kind of data collection could capture the whole picture of food choice decision makings in the participants. Food Choice Map (FCM) interview tool provided such an opportunity to obtain a complete weekly intake and reasons behind food choices. This approach is unique in the literature. Because of this approach, the nutrition intervention was not simply making dietary assessment and deliver education, but to create a personalized, achievable dietary plan with consideration of participants reasons for food choice decision making. A copy of the FCM with agreed changed written on the copy was given to the participant. This copy was served as the diet plan to promote dietary behavior changes. A follow-up dietary consultation was performed at 2 months later to reinforce the recommendations.

### Control group

Participants in the control group did not receive the exercise and dietary interventions. Participants in the control group received standard prenatal care recommended as by the Society of Obstetricians and Gynecologists of Canada. They were also provided with a package of current information on physical activity and healthy eating during pregnancy from the Health Canada [25].

### Data collection

Data on delivery route, maternal weight at delivery room, birth weight and birth weight-related obstetric procedures (induction, forceps or caesarean section) were collected from hospital medical charts by student assistants without knowledge in study design. Diagnosis of gestational diabetes was done by the participant’s attending health care team according to the 2008 guidelines of the Canadian Diabetes Association [26]. Large-for-gestational-age was determined based on birth weight and gestational age as previously described [27]. Pre-pregnancy weights of participants, height and BMI were obtained from the Manitoba Prenatal Care Record. If the pre-pregnancy weight was missing on the record, the weight at the first contact of study participation (less than 10 weeks gestation) was used as pre-pregnancy weight. GWG was defined as maternal weight at delivery subtracts pre-pregnancy weight. EGWG was calculated by subtracting the upper limit of normal weight gain for corresponding pre-pregnancy BMI according to the 2009 guidelines of IOM [7] from the actual weight gain (difference between pre-pregnancy weight and bodyweight at delivery room).

Physical activity levels at the enrolment and 2 months thereafter were assessed subjectively in all participants using a PARMed-X form for Pregnancy designed by the Canadian Society of Exercise Physiology, which was validated previously using peak oxygen consumption [28]. Unfit (physical activity index = 0) during pregnancy was defined as recreational activity <1–2 times/week plus <20 minutes/time. Active (physical activity index = 1) was defined as recreational activity 1–2 times/week, >20 minutes/time or >2 times/week but <20 minutes/time. Fit (physical activity index = 2) was defined as recreational activity >2 times/week plus >20 minutes/time [19].

Food intakes of all participants were assessed using 3-day food records at enrolment and 2 months after the enrolment [29]. The results of self-reported food intake were analyzed using NutriBase 6.0 software containing Canadian Food Database (Cyber- Soft, Inc., Phoenix, AZ, USA).

### Statistical methods

The statistical analyses were performed by a third party. Quantitative data were expressed in mean ± SD. The comparisons for continuous data between 2 groups were conducted using the Student t-test. Categorical data were analyzed using non-parametric Fisher’s exact test. The significant difference was pre-set at p < 0.05.

## Results

One hundred and thirteen participants (56 in the control group and 57 in the intervention group) completed their program and delivered babies before December 31, 2011. All participants in the intervention group met with the dietitian at baseline and at 2 months after. These women attended the group exercise and exercise regularly at home according to the protocol. No withdraw from both intervention and control groups. In the intervention group, 30 women had their pre-pregnancy BMI ≤24.9, and 27 women had their pre-pregnancy BMI ≥25. In the control group, 27 women had their pre-pregnancy BMI ≤24.9 and 29 women had pre-pregnancy BMI ≥ 25. No significant difference was detected in pre-pregnancy BMI, the proportions of First Nations women, or annual family income between the control and intervention groups (Table 1).

**Table 1:** **Demographic and outcome data**

	Pre-pregnancy BMI ≤ 24.9			Pre-pregnancy BMI ≥ 25		
Variables	Control n = 27	Intervention n = 30	P-value	Control n = 29	Intervention n = 27	P-value
Age (years)	29 ± 6	31 ± 3	0.06	32 ± 5	31 ± 4	0.41
Length of intervention (weeks)	0	27.83 ± 5.67		0	26.74 ± 6.17^*^	
Family annual income ($)	54,404 ± 33,689	53,564 ± 24,128	0.91	50,992 ± 23,199	56,772 ± 26,355	0.39
First Nations (number %)	1/27	2/30	0.62	4/29	3/27	0.92
Pre-pregnancy BMI	22.6 ± 1.9	21.6 ± 2.2	0.06	29.7 ± 1.3	29.5 ± 5.1	0.92
Gestational weeks (week)	39.6 ± 0.9	39.7 ± 1.1	0.78	39.8 ± 1.1	39.7 ± 1.3	0.92
Gestational weight gain (kg)	16.23 ± 4.38	12.9 ± 3.72	0.03	14.39 ± 7.05	15.21 ± 7.5	0.26
EGWG (2009 IOM guidelines number %)	10/27 (37%)	3/30 (10%)	0.03	20/29 (69%)	18/27 (67%)	0.67
Birth weight (g)	3,633 ± 555	3,356 ± 474	0.047	3650 ± 481	3,665 ± 506	0.92
Large-for-gestational-age (n %)	3/27 (11%)	2/30 (7%)	0.902	1/29 (3%)	4/27 (15%)	0.13
Gestational diabetes (n %)	0/27	0/30	NS	3/29 (10%)	1/27 (4%)	0.307
Cesarean section (n %)	0/27	0/30	NS	2/29 (7%)	0/27	0.503

Values were expressed in mean ± SD or case/total (0%). P values with underline are statistical significant.

*The p value between the pre-pregnancy BMI ≤ 24.9 intervention group and the Pre-pregnancy BMI ≥ 25 intervention group is 0.49.

In the normal pre-pregnancy BMI subgroups, the amount of GWG was approximately 20% lower in the intervention group compared to that in the control group (16.23 ± 4.38 kg vs. 12.9 ± 3.72 kg, p < 0.05). The rate of EGWG was significantly lower in the intervention group compared to that in the control group (10% versus 37%, p < 0.05). Birth weights of offspring of participants in the intervention group were significantly lower than that in the control group (3 633 ± 555 g vs. 3 356 ± 474 g, p < 0.05). These variables were not significantly different between the intervention and control groups in the above normal pre-pregnancy BMI women. No significant difference was detected in the prevalence of large-for-gestational age baby, gestational diabetes or cesarean section requirement between the intervention and control groups in women with different pre-pregnant women BMI categories (Table 1).

All participants returned food records and physical activity questionnaires at baseline and at 2 months after. At baseline, no significant difference in nutritional intake or physical activity was detected in normal pre-pregnancy BMI women with and without the lifestyle intervention. The lifestyle intervention significantly improved the pattern of nutritional intake in the normal pre-pregnancy BMI participants compared to the control group. Significantly lower daily intakes of total calorie (2 016 ± 496 kcal vs. 2 551 ± 1 044 kcal), carbohydrate (286.3 ± 80.7 g vs. 355.2 ± 147.6 g), total fat (63.1 ± 23.2 g vs. 87.5 ± 41.6 g), saturated fat (20.0 ± 9.5 g vs. 29.52 ± 16.7 g), and cholesterol (225.0 ± 115.9 mg vs. 340 ± 224.9 mg) were detected in normal pre-pregnancy BMI women who received the lifestyle intervention compared to that in the control group (p < 0.03-0.008, Table 2). Among participants with pre-pregnancy BM I ≥ 25, significantly lower intakes of total calorie (1 986 ± 470 kcal vs. 2 258 ± 546 kcal), total fat (65.7 ± 27.1 g vs. 83.5 ± 30.3 g), saturated fat (20.6 ± 10.3 g vs. 27.8 ± 10.6 g) and cholesterol intake (202.0 ± 104.3 mg vs. 305.7 ± 215.2 mg), but not carbohydrate intake, were detected between above normal pre-pregnancy BMI women with and without intervention at 2 months after the onset of the intervention (p ≤ 0.05-0.01, Table 3).

**Figure 2:**
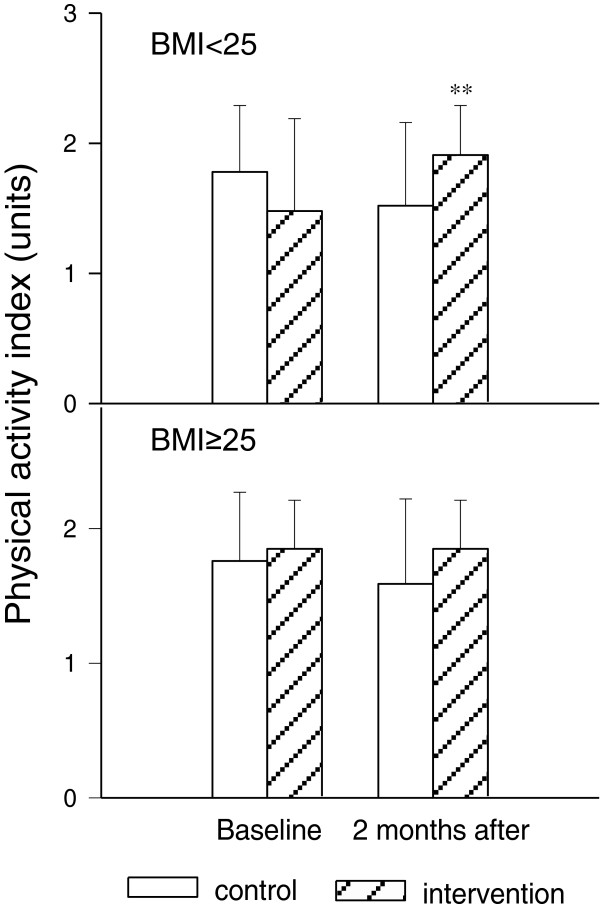
**Effect of lifestyle intervention on physical activity index levels in pregnant women with normal or above normal pre-pregnancy BMI at base line and 2 months after enrolment.** Upper: normal pre-pregnancy BMI (control n = 27, intervention n = 30); bottom: above normal pre-pregnancy BMI (control n = 29, intervention n = 27). Values were expressed in mean ± SD. **: p < 0.01 versus control group with corresponding pre-pregnancy BMI.

At baseline, no significant differences in physical activity level were detected among any group. However, only women with pre-pregnant BMI ≤ 24.9 had significantly higher physical activity units at 2 months after the start of the exercise intervention (intervention group: baseline 1.4 ± 0.81 versus 2 months after, 1.87 ± 0.35, p < 0.05). No significant difference in physical activity was observed in the above normal pre-pregnant BMI group between baseline and 2 months after (control: baseline 1.70 ± 0.61 versus 2 months after 1.56 ± 0.51, Figure 2).

**Table 2:** **Nutrition data of participants with pre-pregnancy BMI ≤ 29.4**

Daily intake	Baseline			2 months		
	Control (n = 27)	Intervention (n = 30)	P value	Control (n = 27)	Intervention (n = 30)	P value
Toatal calorie	2239 ± 654	1982 ± 496	0.12	2551 ± 1044	2016 ± 496	0.01
Carbohydrate (g)	302.4 ± 77.7	272.8 ± 64.1	0.12	355.2 ± 147.6	286.3 ± 80.7	0.03
Protein (g)	90.9 ± 42.8	89.0 ± 27.4	0.85	96.8 ± 40.7	88.7 ± 25.1	0.36
Fat (g)	77.9 ± 30.4	64.4 ± 34.5	0.13	87.5 ± 41.6	63.1 ± 23.2	0.008
Saturated (g)	26.2 ± 12.5	21.17 ± 1.1	0.11	29.52 ± 16.7	20.0 ± 9.5	0.008
Cholesterol (mg)	275.4 ± 182.2	247.6 ± 114.8	0.49	340 ± 224.9	225.0 ± 115.9	0.02

Values are expressed in mean ± SD and analyzed. a: Control versus Intervention at baseline; b: Control versus Intervention at 2 months after enrollment.

**Table 3:** **Nutrition data of participants with pre-pregnancy BMI ≥ 25**

Daily intake	Baseline			2 months		
	Control (n = 29)	Intervention (n = 27)	P value	Control (n = 29)	Intervention (n = 27)	P value
Total calorie	2089 ± 517	2204 ± 693	0.48	2258 ± 546	1986 ± 470	0.05
Carbohydrate (g)	280.1 ± 77.2	303.0 ± 93.8	0.32	294.4 ± 86.7	278.5 ± 64.0	0.44
Protein (g)	91.2 ± 26.1	86.2 ± 25.0	0.46	92.0 ± 26.1	83.1 ± 22.1	0.17
Fat (g)	68.0 ± 23.3	77.8 ± 35.3	0.22	83.5 ± 30.3	65.7 ± 27.1	0.02
Saturated fat (g)	24.7 ± 8.3	22.6 ± 10.4	0.41	27.8 ± 10.6	20.6 ± 10.3	0.01
Cholesterol (mg)	268.8 ± 162.19	193.3 ± 111.6	0.05	305.7 ± 215.2	202.0 ± 104.3	0.03

Values are expressed in mean ± SD and analyzed. a: Control versus Intervention at baseline; b: Control versus Intervention at 2 months after enrollment.

## Discussion

The results of previous studies on the efficacy of lifestyle interventions on gestational weight gain or EGWG in overweight or obese women were inconsistent. Two studies that were similar to our study design were Polley et al. and Wolff et al. [13, 16] Polley et al. reported that education about weight and exercise reduced EGWG in normal weight pregnant women, but not in overweight pregnant women, in a RCT [16]. Wolff et al. described that dietary counseling significantly reduced GWG in obese pregnant women, but did not affect the rate of EGWG between control and intervention group in another RCT [13]. The present RCT demonstrated that pregnant women with normal pre-pregnancy BMI, but not those with above normal pre-pregnancy BMI, had better weight-related pregnancy outcomes including EGWG, GWG and birth weight of offspring following the lifestyle intervention compared to the control group. The results from the present study support the findings that women with above normal pre-pregnancy BMI are relatively resistant to the lifestyle intervention in terms of GWG reported by Polley et al. [13], which is possibly related to the response of the pregnant women to lifestyle education on food intake and physical activity. Normal pre-pregnancy weight appears to be more perceptive to the lifestyle education to improving weight-related pregnancy outcomes during pregnancy. The current study had different results in pregnancy outcomes compared to Wolff’s study. However, total calorie, fat, saturated fat, and cholesterol intake reduction were significantly reduced in the above normal pre-pregnancy BMI group; the pre-pregnancy outcome significance may be detected when the sample size increases.

The nutrition intervention component in this study is unique compared to other nutrition interventions reported in the literature, which used newsletters, group education sessions, personal counselling provided calories and nutrients goals [11–18]. The results showed that all participants in the intervention group made dietary changes regardless with pre-pregnancy BMI. The FCM interview approach ensured a complete review and discussion of a weekly eating pattern. The FCM in-depth interview could explore meanings behind the food choices and is an unique approach in the literature. Results from this kind of data collection could capture the whole picture of food choice decision-makings in the participants. This is a good indicator that the FCM promoted dietary changes with good understanding of the reasons of participant’s food decision making. Women with high pregnancy BMI could have long-term lifestyle habits that influence their food choice, although could be identify through FCM, it might be harder to correct in a limited of time to show significant improvements in gestational weight gain.

Exercise intervention in the literature was either a general encouragement of mild exercise such as walking, or gave verbal or written information on exercise [11–18]. The exercise intervention in this study was a combination of feasible exercise at home and group exercise to strengthen the adherence to the exercise routine. A specifically designed video exercise instruction guided the home exercise which ensured the participant can exercise at the appropriate intensity level on regular basis. This was extremely helpful when the participant could not come to the group exercise due to conflict appointments or weather changes. Weekly group exercise led by a professional trainer helped participants to acquire and validate knowledge and skills for exercise during pregnancy. Group exercise may also help to develop acceptance and adhesion of pregnant women to the healthy lifestyle program. The activity logbook and follow-up visits helped the monitoring of physical activity in participants.

Homogenous recommendation on total calorie intake for normal and overweight pregnant women could be one of reasons for inappropriate GWG and related outcomes in pregnant women with above normal pre-pregnancy BMI. The IOM Food and Nutrition Board published Dietary Reference Intake information for pregnant women in 2006 [23]. Health Canada adapted those recommendations and provided information on key nutrients that are important for maternal and fetal health. The energy requirement recommended for pregnant women with normal pre-pregnancy BMI was 1 900 kcal/day in the first trimester, an extra 452 kcal in the second or third trimester [25]. The extra calorie intake was intended to support fetal growth and development. Our study showed that women in the intervention group with normal pre-pregnancy BMI had a total intake of 2 016 kcal/day in the third trimester, which was close to that recommended by the Health Canada for this group of women. As a result, 90% participants with normal pre-pregnancy BMI obtained weight gain within the recommended limit in the pre-pregnancy BMI category. Women in the control group with normal pre-pregnancy BMI had significantly higher intakes (2 551 kcal/day in average). The majority of the intake in these women also met the total calorie requirement of Health Canada guidelines for pregnant women. This unnecessary level of total intake could partially contribute to increased GWG, EGWG and offspring birth weight in pregnant women with normal pre-pregnancy BMI without intervention.

Calorie recommendation for overweight or obese pregnant women to achieve the IOM recommended pregnancy weight gain has not been defined. Studies in the past had experienced the same difficulties using lifestyle intervention to achieve proper weight gain in pregnant women with high pre-pregnancy BMI women compared to those with normal pre-pregnancy BMI [14]. Two studies [13, 15] specifically targeted pregnant women with higher pre-pregnancy BMI showed successes on weight gain control by setting up meal plans or calorie intake goals. One of the studies reported averages of calorie intake in 1 743 and 1 784 kcal/day for the second and third trimester in pregnant women in the intervention group with no instructed exercise [13]. The other reported an average of 1 900 kcal/day intake with 3-4 times/week walking in pregnant women in the intervention group through pregnancy [15]. These findings suggest that restricted calorie intake could help pregnant women with high –pre-pregnant BMI to achieve recommended GWG. The present study demonstrated that the participants in the intervention group with normal and above normal pre-pregnancy BMI had similar calorie intakes a months after the intervention (2 016 kcal/d versus 1 986 kcal/d). Normal pre-pregnancy BMI, but not above normal BMI, pregnant women receiving the intervention had lower carbohydrate and higher physical activity compared to the control group. The lifestyle intervention reduced EGWG, GWG and birth weight of offspring only in the normal BMI group, but not in high BMI subgroup. It may be speculated that, more intensive intervention to reduce carbohydrate intake and to increase physical activity might be required in order to achieve the goal of normal pregnancy weight gain recommended by the 2009 IOM guidelines [7].

### Study limitations

The sample size of the present study limited the possibility to further divide the study subjects to more detailed subgroups in pre-pregnancy BMI, such as overweight, obese and massive obese, which may weaken the discrimination of the responses from subjects with various intensity of obesity on the lifestyle intervention.

The IOM 2009 guidelines on weight gain recommendations in pregnancy were based on assumptions that a 0.5-2 kg weight gain in the first trimester [7]. Since some participants had no record on pre-pregnancy weight, their earliest weight in pregnancy (<10 weeks of pregnancy) was used as pre-pregnancy weight. This could mildly affect the accuracy of the calculation of total GWG and EGWG.

Although self-reported food records have been commonly used to collect food intake data, there is a possibility that the women with above normal BMI might underreport their intake. It has been reported in the literature that overweight women tended to underreport their daily intake [30]. This could affect the accuracy of the nutrition intake.

## Conclusion and future implementation

The lifestyle intervention program in this RCT effectively reduced EGWG, GWG and offspring birth weight in pregnant women with normal pre-pregnancy BMI, but not in women with above normal pre-pregnancy BMI. Better adaptation to education on food intake and physical activity may contribute to the weight gain control in normal pre-pregnancy BMI women than in those with above normal pre-pregnancy BMI. Future studies may rationalize the level of carbohydrate intake and physical activity for pregnant women with above normal pre-pregnancy BMI, and further explore the effect of enhanced dietary education and physical activity program on GWG in pregnant women with above normal pre-pregnancy BMI. A qualitative study that explores the barriers of women with above normal pre-pregnancy BMI in achieving recommended gestational weight gain may be necessary to understand this population and developing better client-centered education tools.

## Abbreviations

BMI: Body mass index
GWG: Gestational weight gain
EGWG: Excessive gestational weight gain
IOM: Institute of Medicine
RCT: Randomized controlled trial
FCM: Food choice map.
